# Recommendations for designing and analysing multi-arm non-inferiority trials: a review of methodology and current practice

**DOI:** 10.1186/s13063-021-05364-9

**Published:** 2021-06-26

**Authors:** Jake Emmerson, Susan Todd, Julia M. Brown

**Affiliations:** 1 Leeds Institute of Clinical Trials Research, University of Leeds, Leeds, LS2 9JT UK; 2 Department of Mathematics and Statistics, University of Reading, Reading, RG6 6AX UK

**Keywords:** Clinical trials, Multi-arm, Non-inferiority, Multiple testing, Family-wise error, Stepwise adjustment, Simultaneous confidence intervals, Power, Heterogeneous variances

## Abstract

**Background and purpose:**

Multi-arm non-inferiority (MANI) trials, here defined as non-inferiority trials with multiple experimental treatment arms, can be useful in situations where several viable treatments exist for a disease area or for testing different dose schedules. To maintain the statistical integrity of such trials, issues regarding both design and analysis must be considered, from both the multi-arm and the non-inferiority perspectives. Little guidance currently exists on exactly how these aspects should be addressed and it is the aim of this paper to provide recommendations to aid the design of future MANI trials.

**Methods:**

A comprehensive literature review covering four databases was conducted to identify publications associated with MANI trials. Literature was split into methodological and trial publications in order to investigate the required design and analysis considerations for MANI trials and whether they were being addressed in practice.

**Results:**

A number of issues were identified that if not properly addressed, could lead to issues with the FWER, power or bias. These ranged from the structuring of trial hypotheses at the design stage to the consideration of potential heterogeneous treatment variances at the analysis stage. One key issue of interest was adjustment for multiple testing at the analysis stage. There was little consensus concerning whether more powerful *p* value adjustment methods were preferred to approximate adjusted CIs when presenting and interpreting the results of MANI trials.

We found 65 examples of previous MANI trials, of which 31 adjusted for multiple testing out of the 39 that were adjudged to require it. Trials generally preferred to utilise simple, well-known methods for study design and analysis and while some awareness was shown concerning FWER inflation and choice of power, many trials seemed not to consider the issues and did not provide sufficient definition of their chosen design and analysis approaches.

**Conclusions:**

While MANI trials to date have shown some awareness of the issues raised within this paper, very few have satisfied the criteria of the outlined recommendations. Going forward, trials should consider the recommendations in this paper and ensure they clearly define and reason their choices of trial design and analysis techniques.

## Background

Non-inferiority trials are used for determining if new treatments are no more than a pre-determined amount less efficacious than the current standard treatment. They can be particularly advantageous in studies where new treatments provide an alternative benefit to the patient or funder. This includes potentially being less invasive, less toxic, less costly or less time-consuming to administer. It is also becoming increasingly important for trials to be able to run efficiently in order to provide useful and potentially practice-changing information in a timely manner. Currently, one way in which this extra efficiency is achieved is by running trials with multiple arms, where shared control data are compared with the experimental arms. Multi-arm trials can reduce the cost of running studies and decrease the required number of patients to carry out a trial when compared to creating separate trials for each experimental treatment option.

It is not uncommon for non-inferiority trials to include a third arm, further to the experimental and active control arms. In a ‘gold-standard’ non-inferiority design often defined thus within literature [1, 2], the third arm would be a placebo, included to allow a test for assay sensitivity, which ensures that the treatments being compared for non-inferiority are not ineffective themselves. Guidance from the European Medicines Agency (EMA) states that, where ethically allowable, it is preferable for a placebo arm to be included in non-inferiority trials [3]. It is less common for non-inferiority trials to include multiple experimental arms than in a superiority setting, despite the potential advantages of doing so.

There are a number of situations in which non-inferiority trials with multiple experimental arms could be, and indeed have been (see the “MANI trials in practice” section), useful. In disease areas where different viable treatments exist, it would be preferable to test multiple different (potentially new) treatments against one another or against a common control simultaneously if any of the arms are preferable in terms of their toxicity or cost. A trial may wish to test various doses of the same treatment or different dosing discontinuation schedules in order to ensure they still provide an acceptable outcome to the standard treatment schedule. The key difference between these scenarios is the relatedness of the treatment arms and how the hypotheses for such trials may be set up. Both scenarios are of interest within this paper.

A common issue that can arise when carrying out analyses on any trial with multiple experimental arms is potential inflation of the family-wise error rate (FWER), that is, the probability of making at least one type I error from a set of multiple comparisons. When carrying out multi-arm non-inferiority trials, multiple comparisons are made between treatment arms, either between one another or against the control treatment(s). It is important in this case that we ensure the FWER is controlled in order to reduce the chance of making erroneous claims.

This paper investigates the methodology and current conduct of frequentist fixed sample size trials with multiple experimental arms including either an active control arm (or arms) or a placebo arm, or both, where the primary and/or key secondary hypotheses are analysed in a non-inferiority framework. These will hereafter be referred to as multi-arm non-inferiority (MANI) trials. In order to do this, separate searches were carried out to identify literature outlining methodological issues and required considerations for MANI trials and to find examples of where such trials have been carried out in practice.

The aims of this manuscript are to summarise the statistical concerns raised in the literature around running MANI trials and to assess whether or not these considerations are addressed in practice, looking at current and past trials. This will be done by first identifying the key statistical issues involved in designing MANI trials and the considerations required when addressing these issues, before evaluating and comparing the methodological approaches that can be used when analysing MANI trials. The first section will provide a high-level outline of the statistical issues found with reference to some of the available methods for addressing them. These issues will affect both the design and analysis phase of MANI trials but should all be considered within the design phase as part of a statistical analysis plan for a trial. In the second section, the conduct of current and past MANI trials will be assessed and summarised in order to show whether the issues raised are being addressed in practice. The research is brought together to give recommendations for how MANI trials can be carried out in practice and which methods are most appropriate to implement in different trial scenarios.

### Literature search methodology

A comprehensive literature review was conducted to obtain and analyse all current literature regarding statistical methodology and design considerations required when conducting MANI trials and all current and previous MANI trials that have been carried out. Search terms were developed for the following major electronic databases: MEDLINE (Ovid), EMBASE (Ovid), Science Citation Index (Web of Science) and the Cochrane Library (Wiley), each from inception. The search terms are provided in Appendix A. The first search was conducted in February 2020 and auto alerts were set up to ensure further publications released prior to publication were not missed. Additional publications were identified by searching references and citations of useful literature.

**Fig. 1:**
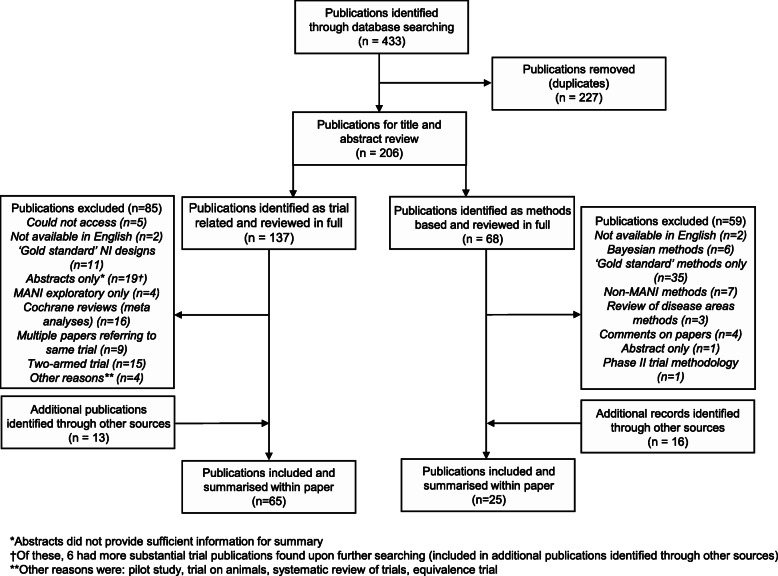
Literature search flowchart

From the original search, publications were split into methodological literature and trial literature based on title and abstract review, whereupon papers were read in full to assess whether they were suitable for inclusion within the review. The details of the search can be found in Fig. 1.

In addition, an assessment of regulatory, guidance and review documents on non-inferiority trials was carried out to identify further possible trial considerations required that could be relevant. These mostly included guidance documents and reviews from groups of experts.

Methodological papers were considered in the MANI setting and for ‘gold-standard’ non-inferiority trials (as defined above); these were assessed to identify whether they were also applicable in the MANI setting and whether and how the methods changed when doing so. If papers only considered methods for ‘gold standard’ trials, they were excluded. Publications giving general guidance around non-inferiority designs were also included, as well as papers that gave more general guidance around multiplicity adjustment. Other criteria for excluding methods based papers were Bayesian methods, non-MANI methods (i.e. non-MANI and not ‘gold standard’ design based methods), phase II trial methods and papers where insufficient information was given to add to the review (i.e. wider reviews of statistical considerations in a disease area, comments on other methodological papers and abstracts for which further information could not be found).

When searching for practical examples of MANI trials, it was noticed that while the defined literature search strategies identified the majority of such trials, not all trial publications stated specifically that they were multi-arm designs and thus were not included in the results of the original searches. Only MANI trials, i.e. those involving multiple experimental arms (as described in the introduction), were included in the results presented in this paper; three-arm ‘gold-standard’ non-inferiority trials were excluded, as were trials on non-human subjects. We considered trials without a placebo arm to be eligible. The other exclusion criteria for trial papers were non phase III trials, trials where MANI analyses were exploratory only, two-armed trials or trials where analyses were not non-inferiority, abstracts and larger reviews of several trials (in this case searches were carried out for individual trials within these publications).

## Statistical considerations for MANI trials

This section summarises the statistical issues and methodology found within the literature review. When initially searching the methodological literature, there were a large number of publications found that mentioned multi-arm trials and that mentioned non-inferiority trials, but few were found that considered both within the same setting. Across the four databases searched, 68 papers were found that mentioned methodology within non-inferiority trials with multiple arms, of which, upon further reading, 9 were found to be directly applicable to the scope of this review [4–12]. These papers cover a variety of methodological areas concerning the design and analysis of MANI trials. A further 17 papers were found when searching references and citations of papers found within the review. This larger number of papers, being found from references rather than initial searches, is primarily thought to be due to the FDA and EMA guidance documents not appearing in the initial searches in addition to papers that, while not exclusively written as MANI specific methodology papers, still contain concepts and ideas that can be applied within a MANI framework, whether originally referring to multi-arm trials (superiority or in general) or to two-arm or ‘gold-standard’ (as previously defined) non-inferiority trials.

The next subsections outline and summarise the statistical considerations that need to be made when designing and analysing MANI trials. The need for these issues to be addressed will change on a trial by trial basis. The issues, in some cases, are not unique to MANI trials, but may require an alternative approach or further thought than is required in other types of trial. If not properly addressed, the issues outlined may result in bias being introduced into the trial or inflation/deflation of the type I and type II errors of a trial which can ultimately undermine the ability to form strong conclusions from a trial and trust any statistical inferences made.

### Ordering and structuring of hypotheses

The setting up of hypotheses is a fundamental requirement for any clinical trial; they put the research question into terms that trial data can seek to answer. Non-inferiority trials have the added complexity of setting out a suitable non-inferiority margin for the experimental treatments and multi-arm trials have to consider whether the implications of their hypotheses mean that adjustment for multiple testing is required in order to control the FWER of the trial. While adjustment for multiple testing in clinical trials is not a new concept, there are still issues around the best way to do so when carrying out MANI trials, particularly in the analysis stage (see later sections). If required, multiple testing adjustments can be made either through choice of trial design, through choice of analysis method or both.

The question of whether adjustment is required is an area of strong debate within the literature. The recent CONSORT extension for multi-arm trials stated that it is “a challenging issue” and while advocating that trials should state their reasons for adjusting or not adjusting, they refrain from making explicit statements as to when this should be done [13]. Determining whether adjustment is warranted should be decided appropriately for each trial after reviewing the literature. Some of the potential areas to consider when designing a trial are summarised throughout the remainder of this section along with some of the schools of thought in these areas.

One of the key indicators as to whether multiplicity adjustment may be required when analysing a multi-arm trial with a shared control group is the structuring of the hypotheses. Howard et al. (and references therein) [14] summarise many of the current opinions within the literature around whether adjustment is required in multi-arm trials, stating that most of the disagreement surrounds the definition of a family of hypotheses. This paper is not specific to MANI trials and generally uses superiority trials within its examples, but the ideas can be applied to all multi-arm trials with a shared control group. The authors state that many views are “based on philosophical opinion rather than statistical theory”.

Howard’s philosophy on the subject is that the ordering and nesting of the hypotheses is critical when making a decision regarding adjustment [14]. Their criteria for adjustment are that if hypotheses are used together in order to make a single claim of efficacy and all individual hypotheses have to be rejected in order to reject a global hypothesis, then it is not necessary to adjust to control the FWER; however, it may be necessary to adjust for the increased probability of observing multiple type I errors simultaneously. If the hypotheses do not all have to be rejected to claim efficacy (but they all form a single claim of efficacy), then it is necessary to adjust to control the FWER. If the arms lead to individual claims of efficacy, it is argued that no adjustment is required as this is where the trial is being penalised for increased efficiency over carrying out multiple two-armed trials.

The decision of how to structure the hypotheses in order to best answer a research question is also reflected within the choice of power to be used within a trial. Westfall and Young [15] give a summary of the different possible choices of power and in which trial situations they are appropriate, for both MANI and other types of multi-arm trial. These include the all pair power, which is the probability of correctly rejecting all false hypotheses, the any pair power, which is the probability of correctly rejecting at least one false hypothesis and the per pair power, which is the probability of rejecting a specific false hypothesis (generally the hypothesis of highest interest).

This interest in specific trial arms can also be reflected in the choice of contrast coefficients when comparing multiple trial arms. Contrast tests are a useful strategy to use when assessing multiple doses of an experimental treatment, particularly within dose response detection [16]. They involve applying different weights to trial arms, whether to reflect an increasing dose in arms or to allow comparison of trial arms against one another as well as against a common control. Chang [4] looks at using contrast tests in MANI and multi-arm superiority trials with continuous, binary and survival endpoints and summarises how different choices of contrast tests can affect the power and the overall sample size.

Dmitrienko et al. [17] give a good summary of the different methods of setting up multiple hypothesis tests in multi-arm trials (not specifically MANI) and which methods require multiplicity adjustment. This includes union-intersection (UI) and intersection-union (IU) testing where either only one hypothesis must be rejected for a global null hypothesis to be rejected or that all individual hypotheses must be rejected to do so. They also summarise closed testing procedures and partitioning tests which are more powerful than assessing hypotheses on an individual basis while still allowing the FWER to be strongly controlled. Closed testing procedures, one of the more common methods of adjustment, involve creating a closed ‘family’ of hypotheses, for which every intersection between hypotheses is tested at a local level; hierarchical testing of hypotheses can correspond to simple closed testing procedures. Partitioning tests involve splitting unions of hypotheses into multiple, mutually exclusive hypotheses and testing them individually.

Hasler and Hothorn [5] use UI and IU testing principles in their procedures when assessing different approaches to analysing MANI trials with multiple correlated endpoints. They summarise their preferred analysis approaches for various hypothesis structures and outline where adjustment for multiple testing is required. These structures include global non-inferiority where the alternative hypothesis for every treatment and endpoint must be rejected in order to conclude non-inferiority and testing for global non-inferiority for a treatment group where alternative hypotheses for every endpoint within a single arm must be rejected in order to conclude non-inferiority for that arm.

### Changing hypotheses when carrying out MANI trials

In addition to the initial considerations around the structuring of the hypotheses, it may also be of interest to change the hypothesis of a trial upon observation of a result (preferably having pre-specified this possibility before running the trial). Typically, this is carried out when switching from a non-inferiority test to a superiority test. If this was to be carried out for a single experimental arm and one endpoint and the primary endpoint remains the same, then there is no increase in the possible type I error as the switch corresponds to a simple closed testing procedure [18]. However, the FDA [19] warn that once multiple arms (or endpoints) are included in a trial where non-inferiority and superiority are tested, there can be inflation in the FWER and say that adjustment may be required.

Ke et al. [20] look at the scenario where in addition to switching from non-inferiority to superiority for a primary endpoint, another secondary endpoint is tested for superiority in a hierarchical fashion. They discovered that in this case, whether for a MANI trial or a two-armed non-inferiority trial, the FWER can be inflated and thus a suitable multiplicity adjustment should be made. More specifically, without multiplicity adjustment, “the type I error rate increases as the non-inferiority margin gets larger and inflation is more severe for moderately positive correlation between the two endpoints”. This is addressed in Lawrence’s [10] paper where he develops a closed testing procedure for the scenario described in Ke et al.’s paper that suitably controls the FWER even with multiple experimental treatments. Zhong et al. [12] also address the issue of simultaneously testing for non-inferiority and superiority in MANI trials; their solution for ensuring strong control of the FWER was to implement adjustment methods within the trial analysis itself, rather than using a closed testing procedure within the setup of the hypotheses.

### Choice of non-inferiority margin definition and subsequent analysis method when carrying out MANI trials

The choice of method with which to assess non-inferiority is dependent on whether it is possible to include a placebo arm within a trial. While acknowledging the ethical issues around if it is appropriate to do so (these are documented and summarised within ICH E10 [21]), the EMA recommend that a placebo should be included within a non-inferiority trial wherever possible [3].

Huang et al. [7] outline that when a placebo arm can be included within a MANI trial, there are two options for how to set up the non-inferiority margin, with all analysis methods falling into one of the two groups. These are referred to as the “so-called fraction methods, which formulate the NI margin as a fraction of the trial sensitivity” (see Pigeot et al. for an example [22]) and the approach where “the NI margin is expressed in terms of the difference of the effects of the E (experimental) and R (reference - referred to as control here)”. This is the method applied in Kwong’s papers on hypothesis testing procedures in MANI trials [8, 9] and according to Huang is “more popular in clinical studies”.

In their guidance on selecting an appropriate non-inferiority margin, the EMA [3] recommend that sufficient thought is given as to the exact aims of the non-inferiority trial before a decision is made on the choice of analysis method. They argue against the use of fraction based methods when the aim of the trial is “to show that there is no important loss of efficacy if the test product is used instead of the reference” and say they are only suitable if the main aim of the trial is to show that the experimental treatment is (or would be) superior to placebo. They do not recommend a single best method of analysis, but give guidance on possible considerations depending on the state of the disease area.

If it is not possible to include a placebo arm within a non-inferiority trial, the assay sensitivity of the control arm can only be argued on a historical basis. In their guidance on non-inferiority trials, the FDA [19] outline two ways of approaching the analysis if this is the case: the fixed margin approach and the synthesis method. Both of these methods are within the second group outlined by Huang et al. [7] where the NI margin is given as an expression of the difference between the experimental and control arms. The FDA guidance does not mention the “fraction methods” spoken about by Huang et al. which further illustrates their point that the group of methods which set the NI margin in terms of the difference between the control and experimental treatment are more popular to use in MANI trials.

The fixed margin approach is used as the basis for the hypothesis testing methods for MANI trials seen in papers from Huang et al. [7], Kwong et al. [8, 9] and Zhong et al. [12]. These methods are spoken about at greater length in later sections of this paper and in Appendix B. The synthesis and fraction based methods were only briefly mentioned when talking about the set-up of hypothesis testing in the MANI trial methodological literature.

### Increasing efficiency

A common issue with non-inferiority trials is that they generally require a large sample size, especially in cases where the non-inferiority margin is close to the estimated effect of the control treatment. Kwong et al. [9] explore optimisation of sample size within their paper, with an algorithm that searches numerically for suitable allocation ratios for the experimental and placebo arms that reach the required any-pair power, enforcing that neither can have a greater allocation than the control arm. They then reduce the total sample size until it is no longer possible to reach the required power.

For MANI trials designed to identify the minimal effective duration required from a number of experimental arms, Quartagno et al. [11] advocate modelling the entire duration-response curve and allocating patients to different durations of treatment. They argue that doing this avoids the potential issues caused when selecting non-inferiority margins as well as reducing overall sample size. They also carried out simulations in order to assess the ability of their duration response curves to accurately reflect the true response curve when using different numbers of duration arms, different increments in duration and different flexible regression strategies in the modelling process in order to make recommendations as to their optimal modelling strategy in different trial scenarios.

### Simultaneous confidence interval compatibility with *p* value adjustment for controlling family-wise error rate

While it is possible to adjust for multiple testing within the setup of the trial hypotheses, it may not always be practical or within the frame of interest for the study to do so. In some cases, adjustments will be made at the analysis stage of the trial. There are many methods available for adjusting an analysis to account for potential inflation of the family-wise error rate; however, when considering MANI trials, issues can arise when implementing some of these methods, due to the results which are used to make inferences in such trials.

Superiority trials often make statistical inferences from *p* values while inferences from non-inferiority trials are conventionally taken from confidence intervals (CIs). In a standard two-arm randomised controlled trial where no multiple testing is present, it is easily possible to obtain both a *p* value and a CI for the treatment difference, regardless of the framework of the trial, and the conclusions with regard to efficacy of the treatment will agree. However, when multiple testing is involved, there is less information available on methods of adjusting a confidence interval for multiplicity than there is for adjusting *p* values [2].

In practice, for single-step adjustment scenarios, in the case of *p* values, adjustments are made by first calculating a simple *p* value from a given hypothesis and then comparing it to an adjusted critical value (or by making an equivalent adjustment to the *p* value and comparing it to a standard critical value). This can easily be translated into creating adjusted CIs (whether for treatment difference on a continuous scale, log odds ratio or log hazards ratio etc.) using a standard confidence interval formula with an altered critical value. This can be done for Bonferroni testing and for Dunnett tests (an adjusted version of this method is implemented in Hasler and Hothorn’s paper [5]).

In stepwise cases, that is, for methods such as the Holm or Hochberg procedure where *p* values are ordered and tested sequentially against increasing or decreasing critical values, it is not easy to achieve correspondence between an adjusted *p* value and an adjusted CI, with simple ad hoc methods of creating corresponding adjusted CIs shown to lose overall coverage [23]. Most adjusted CIs are all calculated together and so are more commonly referred to as simultaneous CIs (SCIs). There are contrasting messages in the literature (summarised below) with some saying that for stepwise adjustment methods, SCIs should be avoided while others have made attempts at creating approximations to stepwise *p* value adjustment methods for SCIs such as the Holm, Hochberg and Hommel procedures. These methods will be outlined in further detail in later sections.

A good alternative to the outlined single step and stepwise procedures is to use parametric methods of adjustment such as Dunnett t testing and Tukey’s honestly significant difference [24]. These methods provide the added advantage of taking the correlation of the tests, induced by the shared control group, into account while non-parametric methods usually assume tests are carried out independently of one another and can become more conservative when this is not the case. They are designed for continuous, normally distributed data and operate by adjusting the method to calculate the standard error such that the FWER can be strongly controlled. This allows for the creation of critical values that can be used to calculate SCIs which are less conservative than those created using Bonferroni-based critical values. Tukey’s method has the added advantage of being able to look at pairwise comparisons between treatments while Dunnett’s method is designed only for comparisons between treatment arms and a shared control treatment. Parametric step-up procedures also exist [25] which are even more powerful than the previously mentioned methods but suffer the same issue with lack of correspondence when creating SCIs as the non-parametric stepwise methods.

When looking at multiple testing procedures from a superiority perspective, Phillips and an expert group [26] state that the majority of discussants felt that using unadjusted confidence intervals is preferable when reporting results. It was felt that when choosing a multiple testing procedure a “hypothesis test should take preference” if a corresponding method is not available for calculating an SCI. The reason given is that formal hypothesis testing to establish an effect and creating a CI is different to creating a CI based on a previously established effect from a hypothesis test. For non-inferiority and equivalence studies, the expert group concluded, “compatible simultaneous CIs for the primary endpoint(s) should be presented in all cases”. This is because in these studies, CIs are typically used to make inferences regarding the hypotheses. The authors regard compatibility (that is, the SCI having the same conclusion as the adjusted *p* value) with the multiple testing procedure as “crucial”.

Channon [27] is of the opinion that the conclusion from CIs should always match those of *p* values. Although outlining some methods of creating SCIs, he recommends that step-down multiple test procedures “should not be used in circumstances where the confidence interval is the primary outcome”. This is reasoned using quotes from Hochberg and Tamhane [28] who say, “if confidence estimates of pairwise differences are desired then the only option is to use one of the single-step procedures”. The examples given within the paper are within a superiority setting; however, the comments made about the use of step-down procedures are applicable to other trial settings.

A similar conclusion is made by Dmitrienko [29] with regard to the use of SCIs. He concludes that in practice, it is likely that the sponsor would have to use unadjusted CIs for the treatment parameters rather than SCIs if using a stepwise multiple testing procedure to adjust for multiplicity.

It is possible to create approximate SCIs that closely correspond to results from powerful stepwise *p* value adjustment procedures; however, when attempting to do so, there are implications of a trade-off between complexity and utility. Dmitrienko [29] provides an example of a simple SCI that corresponds to the step-down Holm procedure; however, these intervals are said to be “completely non-informative” with regard to providing information on the parameter values. Efird and Nielsen [30] provide a simple method for calculating SCIs based on the Hochberg procedure for log odds ratios.

In order to improve the accuracy and utility of approximated SCIs, more complex methodology is currently under development. Guilbaud [31, 32] has developed simultaneous confidence regions that correspond to Holm, Hochberg and Hommel testing procedures. Although these methods are in development and will likely only continue to improve, the complexity of some of these methods may limit how often they will be applied in practice. The decision as to whether or not to use SCIs or an alternative method of analysis should be considered and justified accordingly on an individual trial basis.

### Accounting for heterogeneous treatment variances

Another way in which FWER can be inflated or deflated when analysing MANI trials is if treatment variances are heterogeneous. Many methods of analysis and sample size calculations can assume that variances are homogeneous which, while appropriate in some trial scenarios, may not be appropriate for others. Huang et al. [7] outline the effect of inappropriately assuming homogeneity on the FWER and introduce two alternative procedures that account for heterogeneity (these are outlined in more detail in Appendix B). However, these methods use hypothesis tests and it is not made clear whether corresponding SCIs can be created from them and whether they would reach the desired level of coverage. Thus, this may feed further into the discussion around whether it is preferable to base inferences on SCIs or on *p* value/hypothesis test based analysis methods.

### Maintaining sufficient power when strongly controlling FWER

The draft FDA guidance on non-inferiority trials [33] came under criticism from a group of European statisticians who were concerned that there was an imbalance between the recommendations on controlling the overall type I error and ensuring that type II error was also protected [1]. It follows that by strongly controlling FWER, and using conservative tests, there is a possibility that false hypotheses may not be rejected. This is partly addressed by implementing more powerful testing procedures, which enforces the importance of creating suitable SCIs corresponding to powerful *p* value adjustment methods. Hommel and Bretz [34] warn that there is an element of trade-off between increased power and reproducibility for different multiple test procedures so this also needs to be taken into consideration on a case-by-case basis. The other common but often less popular method of increasing power is to increase the sample size.

## MANI trials in practice

In this section, we move on to summarise the current conduct of past and present MANI trials and assess whether the issues outlined in the previous sections are presenting themselves frequently in practice and how well they are being addressed when they arise. Where areas are not being addressed or are not being addressed sufficiently, we will try to identify possible reasons as to why this may be and formulate recommendations on how to improve how issues are dealt with.

### Practical examples of MANI trials

Across the four databases searched, 65 examples of MANI trials have been found to date. The original search presented 137 papers, once repeats were removed. Of these, 85 were removed, the majority of which were papers relating to the same trials or abstracts of trials that were already included (28). Twenty-six trials did not have multiple experimental arms and four either did not test both experimental arms for non-inferiority or only included non-inferiority testing as an exploratory measure. Sixteen publications were excluded as they were Cochrane reviews of multiple trials in a disease area and therefore did not include design details of individual trials. The remaining 11 exclusions were for number of reasons including phase II or pilot studies, trials on animals or publications being inaccessible. Thirteen further trials were found, either from searching for further details on abstracts, through references from other trials and from methodological papers. The breakdown of this search is shown in Fig. 1.

Of the 65 found, 21 had been carried out across multiple countries, with the remainder taking place in a variety of individual countries around the world and within a variety of disease areas. The most common disease was cancer (nine), with breast, lung, pancreatic and rectal included among the MANI trials in this area. Six diabetes trials were found and five trials were found in HIV and pregnancy respectively, with all other disease areas having four or less trials found. Table 1 gives a summary of the MANI trials and some of their key characteristics and conduct. When assessing whether multiple testing was required, this was counted either based on where trial publications had identified and reasoned the requirement for it or where adjustment was judged to be required based on Howard et al.’s recommendations [14].

**Table 1:** Summary of conduct of MANI trials

No. of MANI trials assessed	65
**Trial background and setup**	
No. trials with authors from primarily academic/industry backgrounds	56/9
No. trials with funding primarily from non-industry/industry sources	25/40
No. single centre/multi centre trials	10/55
No. with NI primary/key secondary endpoint	58/7
**Design considerations**	
No. of trials that implemented a fixed margin approach (as outlined by FDA)	61
No. of trials that included a placebo arm	7
No. of trials that implemented a method to optimise sample size	1
**Multiple testing**	
No. of trials where adjustment was required for multiple testing	39
No. of trials where adjustment was made for multiple testing	31
No. of trials adjusted with a closed testing procedure	14
No. of trials implementing Bonferroni adjustment	12
No. of trials implementing other methods of adjustment for multiple testing	5
No. of trials where superiority tested after NI proven	15
**Analysis considerations**	
No. where heterogeneous treatment variance mentioned	0
No. of trials that clearly defined a choice of power	35
No. of trials that used all pair power/any pair power	11/24
**Data type**	
No. trials with continuous MANI endpoint	34
No. trials with binary MANI endpoint	22
No. trials with survival MANI endpoint	9

Of the 39 trials identified as requiring adjustment for multiple testing, according to the criteria set out by Howard et al. [14], 30 (77%) did so. This framework of deciding whether adjustment was required, outlined in the “Ordering and structuring of hypotheses” section, was selected as it is straightforward to assess across trials and was utilised in the creation of CONSORT guidance on the topic. However, as also outlined in the “Ordering and structuring of hypotheses” section, the topic of adjustment and its requirement in trials has many schools of thought and so this is not the only possible option for assessing the requirement. One trial adjusted for multiple testing despite not being identified as requiring it. Of the adjustment methods implemented, 26 were closed testing procedures or Bonferroni adjustment. Some trials included multiple methods of adjustment for separate endpoints, for example, the LEAD-1 study [35] used a closed testing procedure to adjust for the primary endpoint while using Dunnett SCIs for the secondary endpoint. Of the ‘other methods’ of adjustment, three used Dunnett SCIs (not shown in the table as all used closed testing procedures for the primary endpoint), two used the Bonferroni-Holm method with *p* values, two used Tukey’s method of multiple comparisons (one not shown in the table as Bonferroni adjustment used for the primary endpoint), one used a Hochberg based SCI method and one used a Lan DeMets alpha spending function as there were multiple stages within the trial.

Fifteen trials tested for superiority after proving non-inferiority, 11 of these trials implemented a closed testing procedure while four either chose to use Bonferroni adjustment, or chose not to adjust at all. Other trials which implemented closed testing procedures did so when deciding whether to test further arms based on the success of previous treatment arms. One such example of this is the trial carried out by Bachelez et al. [36] where two different doses of an experimental treatment, tofacitinib, against a control treatment, etanercept and a placebo. In this trial, a fixed sequence procedure was outlined where the higher dose of the experimental treatment was first to be tested for non-inferiority against the control, then if successful, for superiority against the placebo. If this was shown, both steps were to be repeated for the lower dose of the experimental treatment before finally testing the larger, then smaller dose for superiority against the control treatment. As it happened, the higher dose met the non-inferiority and superiority criteria against the control and placebo respectively but the lower dose did not meet the criteria for non-inferiority against the control which meant that no further hypotheses could be tested.

Heterogeneity of treatment variance was not considered in any of the MANI trials that were investigated. Trials generally assumed homogenous variances or did not mention the treatment variance within the publication. Only one trial mentioned the use of a dynamic sample size calculation. This was due to a lack of availability of a sample size formula for their endpoint; Kroz et al. therefore utilised a ‘marginal modelling approach for correlated parameters based on general estimation equations’ [37] which was developed by Rochon [38]. They state that due to their underestimation of dropout levels, future trials in that disease area should have larger sample sizes.

Of the trials assessed, only seven included a placebo arm. The reasons for not including a placebo generally were due to ethical issues or recruitment concerns. In many cases, historical data had previously indicated the efficacy of control treatments. The majority of trials chose to implement what most closely resembled a fixed margin approach as outlined within the FDA guidelines. Some did not define an expected effect of the control treatment and only defined a non-inferiority margin, often with minimal explanation as to the decision behind the choice of margin. Of the four trials that are said to have not used it, there was no clear indication of what design they implemented.

### Statistical considerations of MANI trials in practice

Table 1 shows that while current and previous MANI trials deal with some of the statistical issues identified in this paper, there are issues that either rarely arise, or are rarely addressed. In general, there was an indication that trials showed a preference towards assumptions and methods that would keep design and analysis simple. In many cases, the failure to be explicit in providing reasoning around design choices may have been due to a lack of awareness of the issues raised within the “Statistical considerations for MANI trials” section.

The trials identified generally set up their hypotheses in a manner similar to the fixed margin approach outlined by the FDA in their guidance [19]. The majority did not have placebo arms and did not define an expected effect of the control arm. The trials that did use a placebo stated that they used pre-specified non-inferiority margins that were given in terms of the endpoint rather than as a percentage of the control treatment. Despite failing to define all quantities used in the FDA’s definition of a fixed-margin design, these values may have been known and used implicitly when setting a non-inferiority margin in many of the MANI trials and therefore they have been counted as fixed-margin designs. No trials used the fraction-based approaches mentioned by Huang [7]; this is likely due to the requirement of a placebo arm in calculating an overall test statistic, while the fixed margin approach uses a separate assay sensitivity hypothesis which may not require testing if suitable evidence exists to suggest efficacy in the active control arm.

Almost a quarter of the trials included the option to test for superiority once non-inferiority had been concluded The majority of these trials utilised a closed testing procedure in order to adjust for multiple testing. This seems to be a strong and simple approach to follow when superiority is of interest and is clearly far more efficient than carrying out separate trials to test non-inferiority and superiority.

While power was mentioned in almost every trial, almost half did not explicitly state or strongly imply which type was used. Three trials did consider two types of power, looking at power for individual tests before giving an ‘overall’ power. In some cases, it may have been assumed that the choice would either be known or could be inferred.

The closest that any trial came to sample size optimisation was the dynamic sample size calculation summarised in the “Practical examples of MANI trials” section, for which the resultant sample size was seen to provide insufficient power to provide strong conclusions for the trial. This method was implemented due to a lack of availability of a sample size calculation for the chosen testing strategy rather than to reduce the overall sample size. There is potential for sample size optimisation to be useful within MANI trials, particularly where funding is an issue as non-inferiority trials can require greater sample sizes than other trials. However, its utility must be taken into consideration within the context of the trial, taking into account whether it is key to reduce sample size and how confident investigators are in their estimates of treatment effect and variance.

It was noted that there were no trials that mentioned having heterogeneous treatment variances or that mentioned carrying out any kind of assessment as to whether an assumption of variance homogeneity was justified. While it may be possible that all 65 trials were in areas where treatment variances are all homogeneous, it could be considered unlikely that is the case. Without knowledge of the treatments and the disease areas, it is difficult to know whether the assumption of homogeneous variances (either explicitly or implicitly stated within the trial publications) is fair. The publications outlining the available analysis methods mentioned in the heterogeneity section have not been cited in any MANI trial publications to date which could mean that either they are not well enough known by those carrying out such trials or that there has simply not been a need for them to date. Nonetheless, this is an area that may require more careful consideration in trials going forward.

Adjustment for multiple testing was required for more than half of the trials found. While the majority of trials that required it implemented at least one method of adjustment, just over 20% of trials that were identified to require adjustment failed to do so, with none of them providing a reason for not adjusting. This is also the situation with the choice of power, which may potentially be due to a lack of awareness of the potential requirement for adjustment or due to the investigators believing that the reasoning could be inferred. It may be that in cases where multiple doses of the same treatment are being tested, investigators see these as separate hypotheses rather than as part of the same family of hypotheses. If this is the case and each arm was tested in its own right and did not provide an indication of overall efficacy of a treatment, then adjustment would not be required.

When considering adjustment for multiple testing in the analysis phase, the choices of adjustment method reflected the preference to utilise simple, well known methods over more powerful but more complex methods. While many trials used Bonferroni adjustment, there were five other methods of adjustments implemented across nine trials. Some trials chose to implement more powerful *p* value adjustment methods rather than creating less powerful SCIs while others chose to use Dunnett SCIs which are more powerful than Bonferroni adjustment but more complex to understand. While the figures showed a preference for utilising CIs rather than switching to *p* values when analysing MANI trials, the majority sacrificed power to detect non-inferiority to do this.

Although it appears that trial teams prefer to implement simple adjustment methods within the analysis phase, in a similar manner to adjustment within the design process, trials generally do not justify their choice of adjustment method so it is difficult to know the decision process when selecting one. When choosing an adjustment method, it is vital to take the context of the trial into account. If a small number of treatments are being tested, raising the chances of a type I error by a small amount, then the conservativeness of simple adjustment methods such as Bonferroni may be acceptable in order to take advantage of its simplicity to implement. In cases where differences between treatment effects are expected to be small, or only just above the non-inferiority margin, more powerful adjustment methods may be required to ensure that where truly non-inferior treatments exist, they are found. In this case, it would be at the discretion of the investigator as to whether they would prefer the exact results offered by *p* values over the ease of interpretation offered by approximate SCIs.

## Recommendations

In this section, we provide specific recommendations for the issues raised earlier within the paper that must be considered when carrying out MANI trials. We further give examples from previous MANI trial publications where issues have appeared and been addressed appropriately. It is important to note that issues should be considered within the context of each trial and thus these recommendations may not provide a completely exhaustive summary.

### Clear definition of the structure of all hypotheses

It is vital to be clear within the design phase about how the hypotheses of a MANI trial are to be structured. The best choice of structuring is dependent on the aims of the trial and which hypotheses are of the most interest. Carrying out analysis for multiple hypotheses can increase the chance of observing a type I error and this may require addressing if hypotheses are used together to form a single claim of efficacy for a treatment. The decision as to whether or not adjustment is required and, if applicable, whether this adjustment will be done within the design of the trial or when carrying out the analysis should be clearly outlined and reasoned within any trial documentation (e.g. the protocol and statistical analysis plan). It is particularly important to outline reasons for not adjusting if it is not adjudged to be necessary.

If adjustment is adjudged to be required, it can be addressed within the hypothesis setup by implementing a closed testing procedure or similar sequential testing approach where certain hypotheses are only tested upon the rejection of a previous hypothesis. In this case, the order in which hypotheses are to be tested should be considered and specified. This is generally useful when testing different doses or testing schedules of the same treatment in a trial, for example, the trial outlined by Bachelez et al. [36] tested different doses of tofacitinib within a closed testing procedure where the lower doses of the experimental treatment were only tested if the higher doses were seen to be non-inferior to the control.

Where it is of interest to know the results of all hypotheses, then sequential testing procedures are not appropriate to use and adjustment may be required within the analysis phase of the trial. If the treatment arms are not related or the trial hypotheses do not form a single claim of efficacy for a treatment then adjustment for multiple testing may not be required as outlined in the “Ordering and structuring of hypotheses” section.

### Clear definition of the choice of power type

The choice of power type is strongly linked to the choice of hypothesis structure. If all hypotheses are of identical interest within a trial, then the power used should be the all-pair power as outlined in the “Ordering and structuring of hypotheses” section. If only one arm is of interest, then either the any-pair power (probability of correctly rejecting at least one false hypothesis) or the per-pair power (probability of correctly rejecting a specified hypothesis) could be utilised. It is important to be clear in the choice of power or to define multiple powers if they are of interest. The MAGENTA trial [39] provides a good example of defining the two different powers they implemented within their trial sample size calculation. In this trial protocol paper, both the any-pair power and all-pair power is defined (expressed as power for each test and overall power).

### A priori specification of any change in hypothesis type

It is possible to include a change in hypothesis type in a MANI trial; generally, this is testing for superiority once non-inferiority has been proven. The easiest way to do this is to set up a closed testing procedure or similar sequential testing procedure, as is seen in the BRIGHTER trial [40] where for the key secondary endpoint, the experimental treatment was tested for superiority once non-inferiority was concluded, as part of a closed testing procedure. When including a hypothesis change in a trial, it is important to specify this before the trial is carried out in accordance with the Committee for Proprietary Medicinal Products guidance on switching between superiority and non-inferiority [18]. This guidance outlines the general considerations potentially required when switching hypotheses for MANI trials and non-inferiority trials that fall outside the definition of a MANI trial.

### Clear definition of the choice of non-inferiority margin and subsequent analysis method

In the “Choice of non-inferiority margin definition and subsequent analysis method when carrying out MANI trials” section, several methods for defining the non-inferiority margin and subsequent analysis methods were outlined. Trials generally chose to utilise the well-known fixed-margin approach. The CONCENTRATE trial [41] implemented this approach and provided a thorough explanation as to how they had selected their non-inferiority margin based on results seen in previous studies in the same disease area. Yuan et al. [42] also provide a shorter but sufficiently clear definition of their non-inferiority margin and its derivation. This is a perfectly adequate method to use and has the added advantage of not requiring the inclusion of a placebo arm which is not true for all methods. The specially designed MANI versions of the fixed margin approach outlined in the “Choice of non-inferiority margin definition and subsequent analysis method when carrying out MANI trials” section and detailed further in Appendix B may be worth consideration as they can include adjustments for multiple testing and can provide an increase in power for tests. The normal fixed margin approach should be considered the standard for MANI trials with explanation only required if a different approach is chosen.

### A priori specification of the choice of adjustment method for multiple testing (where required) within a MANI trial analysis with reasoning

If it is determined that adjustment for multiple testing is required at the analysis stage of a MANI trial (see recommendations on ordering and structuring of hypotheses for when this may be the case), then the choice for whether to analyse using SCIs or adjusted *p* values lies with the trial team. It may be preferable to implement two types of adjustment in a trial; for example, LEAD-1 [35] implemented Dunnett testing for the primary endpoint of their trial and Bonferroni testing for the safety endpoints, providing clear explanations for each. The decision as to which adjustment methods are implemented should be outlined in trial documents at least briefly, with reasoning given for choice of method if potentially unclear.

One of the strongest available options for carrying out adjustment is to use parametric methods such as Dunnett t testing and Tukey’s honestly significant difference. These methods provide correspondence for *p* values and SCIs which is not true for the stepwise adjustment methods outlined in the “Simultaneous confidence interval compatibility with *p* value adjustment for controlling family-wise error rate” section and are more powerful than the single-step methods of adjustment such as Bonferroni testing, which addresses some of the concerns outlined in the “Maintaining sufficient power when strongly controlling FWER” section. They also take account of potential correlation induced by having a shared control group while many single-step and stepwise procedures assume tests are carried out independently.

The main reasons for which it may be preferable to utilise a single-step method such as Bonferroni over the parametric methods would be simplicity of implementation and a preference towards being conservative when making inferences about treatments. If using a single-step approach, it is preferable to create adjusted CIs as these provide more detail on the uncertainty of estimates and exact numerical values of treatment differences compared to *p* values.

If the context of the trial dictates that stepwise adjustment techniques should be implemented, based on current research, it is preferable to use adjusted *p* values or test statistics and critical values, as outlined in the “Simultaneous confidence interval compatibility with *p* value adjustment for controlling family-wise error rate” section and Appendix B. Although methods for creating approximate corresponding SCIs are available, their complexity added to the availability of alternative methods for creating SCIs means that they may not be deemed worthwhile to implement.

### Consideration of the potential requirement for adjustment for heterogeneous treatment variances

The decision as to whether adjustment is required within a trial analysis for heterogeneous treatment variances should be considered. Its requirement will entirely depend on the disease and treatment area and the confidence for which an assumption of homogeneous treatment variance can be made. If the treatment variances are assumed homogenous, this should be stated with reasoning within any trial publications where treatment analysis will take place, with a full understanding of the implications of falsely making such an assumption. Where the assumption is not appropriate, alterations to the analysis methods should be made that take variance heterogeneity into account such as not using pooled variance estimators otherwise there is a risk of inflation or deflation of the FWER and potential loss of power. Appropriate methods of accounting for heterogeneous treatment variances are signposted in the “Accounting for heterogeneous treatment variances” section and within Appendix B.

### Consideration of the potential use of sample size optimising techniques

This is an optional consideration that can be implemented within the design phase of a MANI trial, potentially after the definition of non-inferiority margin and subsequent analysis method. Non-inferiority trials can require a large number of participants and thus it may be of interest to try to reduce the overall sample size of the trial, potentially with adjusted allocation ratios. Kwong et al. [9] provide some examples of carrying out sample size optimisation in MANI trials in their paper. If carrying out sample size optimisation, it is important to ensure that a sufficient level of power is maintained within the trial.

## Conclusions

In this paper, we have sought to identify some of the statistical issues that can occur when designing and analysing Multi-Arm Non-Inferiority (MANI) trials, in order to provide advice on considerations that should be made and to recommend the most suitable methods to deal with the issues where possible.

From our review of current MANI trials, it is clear that with respect to the issues we identified, the obvious first area for improvement comes in defining and explaining the choices made for the design and analysis of trials in a clear and concise manner. Explanations may be more difficult to include in publications due to word counts, but this can be included briefly, referenced or included in an appendix. This point applies to all considerations outlined within the “Statistical considerations for MANI trials” section.

One issue that is of key importance throughout the methodology is control of the FWER. It presents itself both in the design and the analysis stage of a trial. The design of the trial and structure of hypotheses determine whether adjustment is required in the first place and the actual adjustment process needs to consider choice of method and assumptions made around the data. This is an element that requires reflection on the debate surrounding this topic when considering whether to adjust. It is worth noting that while the review of MANI trials in this paper found that of a number were adjudged to require adjustment, many had not done so, this was based on applying the philosophy of one of the schools of thought in this area and the numbers could have been different had a different philosophy been applied.

There were mixed messages across the literature in some areas, particularly regarding adjustment for multiple testing at the design phase with compatibility issues between SCIs and adjusted *p* values and the potential advantages and disadvantages of using each within an analysis. Further research may be required into the development of SCIs that correspond to common stepwise *p* value adjustment methods, particularly in creating methods where complexity does not potentially inhibit the ability to apply them widely in trial research. However, the parametric methods provide a strong alternative to stepwise methods and are relatively easy to apply.

The number of MANI trials found in the literature search illustrated that while there is certainly a place for such trials in modern research. Their benefits in terms of increasing efficiency in finding treatments that may be more cost effective, easier to administer or safer are clear. Their use could increase in the future, particularly as a greater number of treatments become available in disease areas and endpoints such as quality of life are becoming of greater importance. The reason for their limited implementation to date may be for a number of reasons. It may be that lack of familiarity with the design or lack of available guidance for how to conduct MANI trials discourages investigators from using them, or it may be that general issues around multi-arm trials and non-inferiority trials such as increased sample sizes may discourage funding bodies from financing such trials.

The methodological literature search illustrated that MANI trials are an area where there has not previously been a large amount of research with little definitive guidance available on how to run a MANI trial and on any statistical considerations that need to be made further to the standard considerations for multi-arm and non-inferiority trials individually. The small number of publications about methodology within MANI trials indicates that there may be room for further exploration of the topic. It was the aim of this review to keep the search terms relatively wide in order to ensure that no publications around MANI trials would be missed. However, a potential limitation is the number of publications that were found from looking at references and citations of publications rather than from the database searches themselves. This could indicate that there may have been publications that could have been missed in the search process which could have added to the scope of this paper.

The trials analysed in this paper appeared to prefer simple design and analysis choices and methods which while not necessarily an issue on an individual basis will not always be suitable for future trials. With potential to be applied on a wider basis going forward, it is important that guidance is available so that those conducting MANI trials are aware of any potential issues that they need to be aware of and how to address them. This paper aims to provide a good insight into the issues and provide a good resource to refer to when setting up a new MANI trial.

## Data Availability

Data sharing is not applicable to this article as no datasets were generated or analysed during the current study.

## Appendix A

### Literature review search strategies by database

MEDLINE

1. (non-inferior*) or (non?inferior*)
2. (three arm*) or (four arm*) or (five arm*) or (six arm*) or (seven arm*) or (multi* arm*).mp.
3. 1 and 2

EMBASE

1. (non-inferior*) or (non?inferior*)
2. (three arm*) or (four arm*) or (five arm*) or (six arm*) or (seven arm*) or (multi* arm*).mp.
3. 1 and 2

Web of Science

TOPIC: ((“non-inferior*” or “non?inferior*”)) *AND* TOPIC: ((“three arm*” or “four arm*” or “five arm*” or “six arm*” or “seven arm*” or “multi* arm*”))

Cochrane Library

1. MeSH descriptor: [Research Design] explode all trees
2. MeSH descriptor: [Clinical Trials as Topic] explode all trees
3. 1 or 2
4. (non inferior* or non??inferior*) and (three arm*) or (four arm*) or (five arm*) or (six arm*) or (seven arm*) or (multi* arm*)
5. 3 and 4

## Appendix B

This appendix provides further detail around some of the methodological ideas introduced in some of the MANI specific literature mentioned in the first sections. It was not included in the earlier sections in order to ensure that the extra information did not distract from the primary aims of the paper.

Alternative methods of analysing MANI trials have been outlined and developed by Kwong [8, 9] and Zhong [12]. Their methods involve calculating test statistics and comparing them to critical values that they calculate; single-step, step-up and step-down procedures have been developed that can test for both non-inferiority and superiority (upon proving non-inferiority). These methods more closely resemble *p* value adjustment methods as they involve ordering of ‘most significant’ arms and increasing/decreasing critical values but they do not directly correspond to the stepwise *p* value adjustment methods that have been mentioned, including Holm and Hochberg. These methods work under the fixed margin hypothesis framework outlined in the FDA guidance [19] on carrying out non-inferiority analyses and so generally assume the inclusion of a placebo arm within a trial. They are all designed to strongly control the FWER and are compared in their power to detect non-inferiority and superiority which are generally of a satisfactory level. There is no indication within their papers about how these methods compare to *p* value adjustment methods, however their ability to test for both non-inferiority and superiority within the same framework and their specific gearing towards MANI trials, alongside their simplicity compared to some of the approximate SCI methods, are all positive indicators for the methods. One of the disadvantages of these methods are that making inferences based on test statistics is similar to making them based on *p* values where they are less easy to understand with regard to the magnitude of the effect of a treatment compared to SCIs.

Kwong’s first paper [8] outlined an extension to the fixed margin approach outlined in the FDA guidance which allowed it to be utilised in MANI trials and created appropriate test statistics for the single-step procedure that they outlined. Huang et al. [7] utilised Kwong’s single step procedure as the comparator for the two methods they assessed for accounting for treatment variance heterogeneity when analysing MANI trials. The methods assessed by Huang (both single step) were shown to control FWER well for both homogeneous and heterogeneous treatment variances and maintain a similar level of power to Kwong’s method. These methods were published prior to Kwong’s and Zhong’s papers on stepwise testing procedures and thus far have not been formally compared with the methods from these papers, nor has a stepwise method accounting for treatment variance heterogeneity been formally outlined.

Hasler [6] considered similar areas to Huang’s paper and derives separate test statistics, suitable for homogeneous or heterogeneous treatment variances, for trials that include a placebo. These were created from a fraction-based hypothesis framework and take multiple testing into account where it is required. Hasler also extends to deriving lower SCIs for the ratios of differences before going on to discuss any pair power and allocation optimisation. There are no comparisons made between the Hasler’s methods and any other method and they are not applied to formal examples to test their ability to control FWER.

While the methods outlined by Hasler and Huang have their individual merits, they both rely on the inclusion of a placebo within a trial. There is also a lack of comparisons between these methods and other available methods in order to assess whether these methods truly perform better in the presence of treatment variance heterogeneity. In a trial setting, this variance heterogeneity may not be tested for, either due to prior knowledge of the treatment arms or due to lack of awareness to the issue and its potential consequences. However, Huang’s results regarding the potential inflation of FWER if it is not taken into account would suggest that it is something that should be considered and tested for.

## Abbreviations

CI: Confidence interval
EMA: European Medicines Agency
FDA: Food and Drug Administration (US)
FWER: Family-wise error rate
IU: Intersection union
MANI: Multi-arm non-inferiority
NHS: National Health Service
NI: Non-inferiority
NIHR: National Institute of Health Research
SCI: Simultaneous confidence interval
UI: Union-intersection
